# “One of My Basic Necessities of Life Is Work. That’s Just Broken Away.”—Explorative Triangulation of Personal and Work-Related Impacts for Supervisors and Disabled Employees in German Social Firms during the COVID-19 Pandemic

**DOI:** 10.3390/ijerph18178979

**Published:** 2021-08-26

**Authors:** Ann-Christin Kordsmeyer, Ilona Efimov, Julia Christine Lengen, Volker Harth, Stefanie Mache

**Affiliations:** Institute for Occupational and Maritime Medicine (ZfAM), University Medical Center Hamburg-Eppendorf (UKE), Seewartenstr. 10, 20459 Hamburg, Germany; i.efimov@uke.de (I.E.); j.lengen@uke.de (J.C.L.); harth@uke.de (V.H.); s.mache@uke.de (S.M.)

**Keywords:** COVID-19, occupational health, SARS-CoV-2, social enterprises, social firms, working conditions

## Abstract

Social firms are located on the general labor market and employ 30–50% of severely disabled people. Findings on personal and work-related impacts for employees and supervisors during the COVID-19 pandemic are not yet available and will be investigated in the present study. Using the approach of a method triangulation, focus groups with employees and individual interviews with supervisors of several social firms from the North of Germany were combined and collected in parallel. Between July and November 2020, 16 semi-structured telephone interviews with supervisors and three focus groups with 3–6 employees each working within the same team (14 employees in total) were conducted. Both formats were recorded, transcribed, anonymized, and analyzed by using Mayring’s qualitative content analysis. Because a large proportion of the employees and supervisors interviewed worked in the gastronomy sector, they were particularly affected by the “restriction of social contacts” beginning in March 2020. Hygiene and distance regulations were implemented and personnel planning and distribution of work were adapted. Challenges were raised for employees with disabilities due to the implementation of hygiene and distance regulations, a sudden loss of work, lacking routines, additional work, a lack of movement, social contacts and financial challenges. Both employees and supervisors reported fears of infection, conflicts, additional work and fears of job loss. Additionally, supervisors dealt with less staffing, challenges in detaching from work and a strained economic situation. Overall, new insights were gained into the work-related impacts for employees and supervisors in inclusive workplaces during the current COVID-19 pandemic but further research on health-promoting structures is needed.

## 1. Introduction

SARS-CoV-2 (severe acute respiratory syndrome coronavirus type 2) is a new coronavirus that was identified as the cause of COVID-19 (coronavirus disease 2019) in early 2020 and spread throughout the world ever since [1]. To contain the spread, various measures have been initiated in Germany. In March 2020, the federal and state governments agreed on a comprehensive “restriction of social contacts” including interventions for social distancing. Restaurants and numerous other companies were closed. In May 2020, a gradual reopening of public life began with stores, gastronomy, other services, and cultural institutions allowed to gradually reopen under hygiene and distance regulations. In October 2020, a partial lockdown was again decided, which became effective in November 2020. Social contacts were again to be reduced to a minimum and staying in public was restricted to small groups. Numerous facilities were closed again, such as cultural, gastronomy and service establishments. In December 2020, infection control measures were further tensed as of January 2021 due to continued high infection rates [2]. The background to the measures were persistently high infection rates, but also new virus variants identified since December 2020 [1].

For people with disabilities, their families and workplaces, those measures to contain the spread of the COVID-19 pandemic brought severe changes in their daily lives. Above all, disabled individuals are exposed to more severe outcomes when being infected with SARS-CoV-2. More detailed, severe courses of the disease are observed more frequently in people with Down syndrome (trisomy 21), certain pre-existing conditions, e.g., of the cardiovascular system, chronic lung diseases, chronic kidney and liver diseases, psychiatric diseases (e.g., dementia), diabetes mellitus (diabetes), cancer or those with a weakened immune system [1]. Other risk factors include advanced age, male gender, a smoking status or obesity [1]. Dobransky and Hargittai [3] gained insight into a comparison between people with a disability and those without, observing that 64% of those with a disability were at risk of a severe course of COVID-19 in comparison to under a third without disabilities [3]. People with disabilities were also affected by further social participation inequalities before the onset of the pandemic which deteriorated during the current pandemic, such as social isolation or economic challenges [4].

One possibility of employment is represented by social firms, as they provide a high employment rate of people with disabilities (30–50%) (§ 215, Book Nine of the German Social Code (SGB IX)). In general, social firms are characterized by a heterogenic group of employees including severely disabled people with mental or intellectual disabilities or those with a severe physical, sensory or multiple disability (for further definition of social firms see [5] and analysis of working conditions [6]). In the current state of research, a positive impact on health-related outcomes of its employees such as social participation, self-confidence, well-being, quality of work life, changes in health behavior or reduction in hospital visits was evaluated [7,8,9,10,11,12,13,14], wherefore an economic survival of social firms in the current pandemic crisis is particularly important for the target group [15]. However, recent experiences show severe impacts on the economic situation due to the COVID-19-pandemic. Current survey results from 2020 among 335 social firms indicate that significant sales declines were recorded particularly in the catering (mass caterings, restaurants, cafes, bistros) and hotel sector, as well as in education and entertainment. Retail, healthcare, laundry and cleaning sectors were also affected by smaller declines in sales. Furthermore, most companies resorted to short-time work (58.8%, *n* = 284) (transitory reduction of working hours and wage due to a shortage of orders; § 95, Book Three of the German Social Code (SGB III)), overtime was reduced (59.9%) or jobs were relocated to other business areas (22.5%). More dramatic effects were described by 14.4% of participating companies who had to part with employees: 8.8% said they had to part with employees with a disability, 6.3% had already dismissed employees. A total of 62.7% had taken advantage of short-time allowance, as well as other subsidies and financial support services (52.1%) [16].

From other settings, such as sheltered workshops or residential facilities, additional impacts on the daily lives of persons with disabilities were also reported in the current state of research such as lacking daily routines due to discontinuation of work, which resulted in different daily structures as well as reduced social contacts, e.g., with colleagues [17]. The situation was also aggravated by a loss of appointments fostering structure and security such as leisure activities (e.g., shopping or sports) and a loss of contacts with professionals in education, psychology, psychiatry or medicine and other therapy options. In general, a great diversity of the needs of people with intellectual disabilities and their coping strategies with the COVID-19 pandemic became evident as stated by participants without disabilities. Impacts on well-being and health, like emotional upset, fears and anxiety, and an increase in depressive symptoms were reported for people with disabilities by mental health professionals [17,18] as well as social withdrawal, restlessness or aggressive behavior [19]. In addition, uncertainties and helplessness were explained by participants with and without disabilities, due to uncertainties about the further course of the pandemic and resulting preventive measures. However, positive aspects were also mentioned in some cases, reporting a deceleration of life at the beginning of the pandemic [17]. Supervisors faced job demands and feelings of stress due to a flood of information when implementing new measures for infection prevention at work, extending working hours, as well as conflicts between work and family dimensions [17].

Results on the consequences of the current COVID-19 pandemic on the lives of employees and supervisors of social firms are missing and only comparable in a limited way to those from sheltered workshops or residential facilities. To ensure participation and equal rights to safe and healthy working conditions for people with disabilities also in times of the current COVID-19 pandemic based on the UN Convention on the Rights of Persons with Disabilities (Article 9 and 27) [20] an analysis of the current work situation is necessary for social firms. Therefore, two main research questions were addressed:

1. What work-related changes were reported in social firms during the COVID-19?
2. What personal and work-related impacts on social firms’ employees and supervisors were outlined during the COVID-19 pandemic?

## 2. Materials and Methods

### 2.1. Study Design

To examine current personal and work-related impacts of employees and supervisors in social firms during the COVID-19 pandemic, different qualitative methods were triangulated in order to adopt different perspectives on the depicted research questions and thus achieve an expansion of knowledge [21]. Due to a lack of research in this field, a qualitative approach was chosen to examine this specific occupational setting [22]. Focus groups with employees with disabilities and individual semi-structured interviews with supervisors from several social firms from the North of Germany were considered and collected in parallel. These qualitative research methods were used to gain an in-depth understanding of work organization and employees’ and supervisors’ personal and work-related situation during the current COVID-19 pandemic [23,24]. Between July and November 2020, 16 semi-structured telephone interviews with supervisors and three focus groups with 3–6 employees each working in the same team (14 employees in total) were conducted. Focus groups were considered to be the most appropriate and confidence-building method instead of interviews (as it was done for supervisors). The chosen method is particularly suitable for examining people’s different subjective perceptions, experiences, and opinions [25]. A personal relationship existed neither with supervisors nor with its employees beforehand. Interviews with supervisors were conducted and analysed by A.-C.K., a female health scientist (M.A.). Focus groups with employees were conducted by J.C.L. (M.Sc.) and A.-C.K. Data analysis of the focus groups was conducted by J.C.L. and I.E. (M.Sc.). All concerned authors could draw on previous experiences in qualitative research and were employed as research associates.

### 2.2. Participant Selection

All participants of the study were selected by using a convenience sampling approach. Employees were eligible for participating in a focus group if they met the following criteria: (1) a severe disability; (2) a paid job in a social firm (according to § 215, Book Nine of the German Social Code (SGB IX)); (3) older than 18 years and (4) proficiency of the German language. Inclusion criteria for the semi-structured interviews with supervisors were defined as: They (1) had to work at least 18 h per week as a German-speaking supervisor in a social firm (according to § 215, Book Nine of the German Social Code (SGB IX)), (2) had to be at least 18 years old, (3) for at least half a year with (4) direct contact to employees with disabilities. Overall, two supervisors disregarded repeated invitations to the study mainly due to time pressure maintaining day-to-day business operations or as a result of the COVID-19 pandemic.

### 2.3. Data Collection

On the one hand, one focus group was conducted at the workplace of the participating social firm and two focus groups were conducted in conference rooms outside the companies adhering to strict hygiene and distance regulations. Researchers started the focus groups by explaining the study aim, introducing into the conversational rules and code of conduct [25]. At the end of the focus group, a short questionnaire was distributed to all participants in order to collect demographic data. For the focus groups a semi-structured interview guideline (Table 1) with open-ended questions was designed to guide the moderator, to focus on the research questions, to ensure comparability of the focus groups and to increase reliability [23,25]. The comprehensibility of the interview guideline in terms of plain language was consulted and agreed upon with a cooperating provider of health promotion services for people with disabilities.

On the other hand, supervisors were interviewed via telephone at home or at their workplace increasing a flexible and practicable approach during the COVID-19 pandemic. Therefore, participants were instructed to seek a quiet place during the interview without any disruptions. One supervisor was interrupted by employees at work. Beforehand, both employees and supervisors were informed about the study (objectives, methods, analysis, and data protection regulations) and gave written consent. For the interviews with supervisors a semi-structured interview guideline was used (Table 1) based on Witzel’s problem-centred interview technique (PCI) including socio-demographic data at the end [26]. Additional postscripts were conducted (content of the conversation, non-verbal characteristics, thematic emphases and thoughts on interpretation). For both ways of data collection, participation in the study was voluntary and data were collected in German. Each interview guideline was tested beforehand to ensure practicability.

Focus groups were audio recorded and lasted approximately 78:06 min (range: 01:09:11–01:25:03). Interviews with supervisors lasted on average 46:19 min (range: 00:30:19–01:11:00). Neither repeat interviews nor focus groups were conducted, transcripts were not returned to participants for comment or correction purpose and feedback on the results was not collected.

### 2.4. Data Analysis and Reporting

Audio data was transcribed verbatim and anonymised based on the transcription rules of Kuckartz [25,27]. The transcripts of focus groups were examined by J.C.L. and those of supervisors by A.-C.K. Both authors used the software MAXQDA Analytics Pro (version 12, VERBI GmbH, 2016) for encoding. After encoding the first audio data, two researchers (A.-C.K. and I.E.) reviewed the results and discussed sub-categories and associated text segments. The following audio data was encoded by one person only and in the case of doubts on text segments or sub-codes consensus was reached in regular project meetings. Qualitative data from both formats were analysed using Mayring’s qualitative content analysis [28]. Main and sub-categories were developed by two researchers (A.-C.K. and I.E.) in an inductive way. The data transcription and evaluation was carried out without involving the interviewees. Quotes of all stakeholders were translated into English and presented to illustrate the findings. Overall, the COREQ-checklist (consolidated criteria for reporting qualitative research) provided a basis for reporting the results (see Supplementary Materials).

### 2.5. Ethical Considerations

All participants signed an informed consent form, which was additionally handed out in plain language at the beginning of each focus group. Written and oral information was provided to all participants on data protection, the confidentiality and anonymity of study results. The study was conducted in accordance with the Declaration of Helsinki and approved beforehand by the Ethics Committee of the University Medical Centre Hamburg-Eppendorf, Germany (LPEK-0051).

## 3. Results

### 3.1. Characteristics of the Study Population

As Table 2 presents, most of the 30 study participants were male (68.75% of supervisors and 50% of employees) and between 31 and 65 years old. Most participants (*n* = 17) reported more than 3 years of work experience in the current social firm. Supervisors were responsible for 11 employees on average (range 5–25 subordinate employees). Overall, participants were engaged in food and beverage service activities, services to buildings and landscape activities, printing and reproduction of recorded media, office administrative, office support and other business support activities or wholesale of bicycles and their parts and supplies (according to [29]).

### 3.2. Work-Related Changes in Social Firms during the COVID-19 Pandemic

#### 3.2.1. Sector-Specific Economic Changes Due to the COVID-19 Pandemic

Social firms were mostly closed between March and May 2020 for eight weeks. The closure was highlighted as very abruptly occurring within two days. Some of the supervisors maintained a bit of back office work, organizational tasks as well as, e.g., repair work on a small scale.

“*Well, we had officially closed the store for a month. Then I continued to work here, did a little back office, what we were allowed to do. So we continued to do repair work, and these repair jobs were just on a small scale.*”

(Supervisor #11, male)

During summer and fall 2020, the order situation in most participating social firms normalized again, although not to its full extent yet, as canteens, for example, remained closed. In other social firms operating in the event management sector, it was reported that many clients had canceled their venues.

“*That will be the problem that many companies have canceled us, which follow the instruction that they are no longer allowed to travel.*”

(Supervisor #12, female)

Referring to economic outcomes, decreased sales were observed in summer and fall 2020. In most social firms, too little work was available for all staff and this way employees noticed that the enterprise did not do well, despite all subsidies.

“*Yes, for us it was of course a disaster […]. So we […] are down to five percent turnover. I actually put all the employees on short-time. One hundred percent. Because I was all alone here the whole time. […] And I just did that alone, because it wasn’t profitable here. And that was really the first few weeks, we thought it was going to be really bad here.*”

(Supervisor #16, male)

Furthermore, it was described that business developments, company outings or certain training courses could not take place for financial reasons or had to be cancelled due to the pandemic.

“*You can’t develop because the finances aren’t there.*”

(Supervisor #16, male)

In contrast, an economic upturn was observed by social firms of certain sectors, such as the bicycle sector, and supervisors referred to positive stress and improved sales. However, their employees worked more over a period of three or four weeks.

“*Especially after the crisis when there was a boom here, of course everyone worked more, but then that goes on, I say, three maybe four weeks and then you have to say, so watch out people, you now have twenty, thirty more hours here.*”

(Supervisor #8, male)

In the companies that were affected, lots of inquiries were reported as well as more sales and more extensive repair orders. Therefore, no economic losses were noticed after one month lockdown. In fact, employees had to be forced to take a break at work after reopening.

“*Sometimes it is difficult, you also have to force the employees sometimes to say, so you go now in the break, because just now after the opening, reopening, we had such a strong rush in the bike stores that the queue outside did not break off, so to speak a permanent processing was necessary.*”

(Supervisor #8, male)

In the manufacturing sector, social firms performed well at the beginning due to major customers and the printing of flyers, at a later stage customer inquiries decreased. In the cleaning sector, more strict cleaning schedules were introduced.

“*At the beginning we had a lot to do, […] it’s clear, then they needed a lot of flyers, posters, etc., so we had a lot to do from March, April onwards.*”

(Supervisor #15, male)

“*So our department has not really been affected by this in terms of work, we have continued to work quite normally and we were now as a cleaning department not so greatly affected by the whole story, on the contrary we had more effort, we have received more requests, we had to carry out more disinfecting cleaning, so for us it was actually more effort actually.*”

(Supervisor #6, male)

#### 3.2.2. Short-Time Work during the COVID-19 Pandemic

Most participants reported the introduction of short-time work for its employees (mostly 100 or 50 percent) and also supervisors were partly on short-time work or on call in home office, respectively.

“*I actually put all employees on short-time work. One hundred percent.*”

(Supervisor #16, male)

The process of applying for short-time work was stated as a challenge due to lack of experience and combination options with personnel programs.

“*Applying for short-time allowance, we’ve never done all that before and no, and that, that was then also partly not possible with the personnel programs that are used to plan in this kind of short-time allowance.*”

(Supervisor #8, male)

Partly, short-time allowance was topped up for employees, so everyone got the same salary as before. Nearly one year after the outbreak of the pandemic, the situation had improved considerably, however, there were still employees on short-time work.

“*Now the situation is much better, there are still employees on short-time who are taking turns here and we have introduced duty rosters […] I myself was on short-time […], but now I’m not on short-time again, because we’ve noticed that there’s a lot to do.*”

(Supervisor #13, female)

Furthermore, grants of varying amounts were applied for from local authorities or German funding organizations.

“*Therefore we have also naturally subsidies in different levels.*”

(Supervisor #12, female)

#### 3.2.3. Implementation of Hygiene and Distance Regulations during the COVID-19 Pandemic

Hygiene and distancing regulations were described as largely accepted among employees, supervisors and also guests adhering to wearing a face mask. Employees in social firms were immediately provided with face masks. Supervisors saw themselves as a role model (e.g., in terms of not shaking hands or hugging and wearing face masks).

“*The employees have also done very well, we have immediately provided them with face masks, we have made distance bands, […] we have set up disinfectants.*”

(Supervisor #1, female)

“*Even if I am smiled at here and there by my colleagues or told that I am being overcautious, but I only go into every department with a mask, I try to keep the distances, I always try to ask people, no, don’t hug, don’t shake hands, don’t do it, I don’t do it myself either, and of course I also try to set an example.*”

(Supervisor #3, male)

In general, face masks were used at the workplace, but not at fixed workplaces. The teams were partly divided up so that in case one person got sick, the whole team could be replaced and business operations could be continued. Additionally, distance marks were introduced, e.g., on the floor or by means of Plexiglas, the minimum amount of people for each workplace was defined, disinfectants were set up, and surfaces were cleaned on a regular basis. In fact, high hygiene standards were already in place in social firms, e.g., in the gastronomy sector as employees had to disinfect hands when they came from outside.

“*So here were, always had high hygiene, standards for hygiene, like disinfection of hands […], so they have always had to do it, so even if they came from outside, So it has not changed much […], because we are fewer people, you can adjust the distances better in the kitchen, or try to keep the distances in the kitchen, but we also have two teams, if someone is infected, that then the other team could step in, so that fits everything.*”

(Supervisor #10, male)

When introducing new measures for infection prevention, supervisors met with all employees at the beginning and explained what protective measures were available. Employees were outlined as very cautious during the implementation of hygiene and distance regulations, e.g., if someone felt uncomfortable.

“*We have all met together once with the distance of course, which was necessary, I have explained to them once briefly, shown everything, respectively what protective measures we have from face masks to gowns to overalls, even if you did not need that, I have nevertheless provided and shown everything once, with disinfectants, what we have and then asked again if anyone feels uncomfortable with the whole story and whether I should relocate them, elsewhere means then in the case where you have no contact with other people.*”

(Supervisor #6, male)

In some cases, additional support and training through collaboration with company physicians for individual social firms was mentioned.

“*And of course we have […] a company doctor, who comes around or has also just done training courses now on Corona.*”

(Supervisor #8, male)

#### 3.2.4. Adaptations of Personnel Planning during the COVID-19 Pandemic

Due to short-time work and new hygiene and distance regulations, duty rosters were introduced in social firms and shift systems were redesigned so that employees were always scheduled as fairly as possible and that everyone was able to work at some point.

“*We have introduced duty rosters, have made shift systems so that we can always use the people as fairly as possible, that everyone works sometimes and sometimes not.*”

(Supervisor #13, female)

Some employees were not returned to work in summer or fall 2020, especially when working in outsourced workplaces of sheltered workshops, because of pre-existing health conditions.

“*There are still employees who have not yet returned, (…) because a risk analysis has to be made for them, if they have any previous illnesses or so, then it is checked beforehand whether they are allowed back at all and if that is questionable, in case of doubt they are not allowed yet.*”

(Supervisor #5, male)

In some social firms, employees were relocated to other workplaces such as cafés or driving services. Offers were adapted, e.g., selling canned or defrosted food and new ideas and concepts were developed, such as a snack bar:

“*Then they move out in cafes or also driving service. So we try others, we ask, what can you imagine and then we try to employ them somewhere else again.*”

(Supervisor #1, female)

Additionally, supervisors were partly reassigned to other workplaces during the pandemic.

“*The (name of the department) is closed and now at the moment I jump sometimes between the (name of other department), and the other (name of another department).*”

(Supervisor #7, female)

#### 3.2.5. Cooperation and Distribution of Work during the COVID-19 Pandemic

It was reported that regular meetings continued once a month or every two weeks while maintaining the required distance and also taking into account sign language interpreters. In this way, staff were kept informed, new colleagues could be introduced and problems solved. Supervisors provided employees with forms and new information on the pandemic situation at the workplace, which were printed and distributed. Other larger meetings with all employees were partly suspended. Partly, a closer collaboration was reported between supervisors before the pandemic.

“*So we have a meeting every two weeks now in the Corona time, where we sit down with the kitchen and the service and note everything that has arisen in terms of problems, that is, are there problems with staff, […] do we need more staff or is the work manageable with the small groups that we have right now, is something broken, has something to be maintained.*”

(Supervisor #4, female)

In general, during the pandemic, more time was needed to discuss distribution of work tasks, as employees worked on different days during the week and not everyone was able to take on all tasks. Prior to the pandemic, the team briefly came together during the day and discussed what was needed to be done on that day and by whom. In the social firm, special attention was paid to the implementation of changes at work.

“*If I have an order and I have someone who cannot do the job for some reason, then I have to structure the week somehow so that I then put it on another day. And then, during that time, I had to talk about it a lot, a lot more. Before, every now and then we would just kind of get together briefly on the day and say, ‘Look, this and that has to get done. Who’s going to do that?’ And so and then we would have/have that pushed to each other. ‘Okay if you do this, then I’ll do this and in return I’ll take care of something else.’*”

(Supervisor #14, male)

“*So, we try to keep those, the changes within the operation as small as possible if they have to go quickly. If they don’t have to go quickly because we have a new idea on how to maybe do something fundamentally different, then we try to bundle that and change everything at once. Those are the two options we have. Because if we gradually kind of come up with a little change every three weeks, then we don’t have/then this workplace doesn’t provide stability anymore. If we overturn everything once it’s new for everybody. And then people feel safe again because everybody around them is in an insecure position too.*”

(Supervisor #14, male)

Additionally, exchange of information during the pandemic did not work for all those affected, as employees never met completely and elaborated workflows were suspended, requiring supervisors to communicate information to employee group several times.

“*But that meant for me, because I worked full time all the time, that I always had to explain everything two, three times. That was actually the worst part of the whole story for me in this position: that everything beforehand that you had worked on, on workflow/that was all, all thrown over. […] And then it was up to the few who were permanently there to take care of it. Things that actually didn’t need to be taken care of for months, because they were already taken care of. That was the worst part of the whole story.*”

(Supervisor #14, male)

In terms of costumer contact, some supervisors reported that employees were not able to be assigned to be in contact with them because of their increased risk of being seriously ill if infected with SARS-CoV-2.

“*Due to the fact that we now have this pandemic and the requirements that we have to fulfill here, hygiene protection, etc. pp., I can’t use one of my employees at all in the customer/, I can’t use him, because he belongs to the high-risk group and then […] I have a colleague with a stress disorder, with whom I just have to be careful how much customer contact one can expect him to have.*”

(Supervisor #11, male)

Further challenges in communication with customers were described including a stricter communication because of customers’ social needs, which was more difficult to apply for employees with disabilities.

“*Yes, for example that you do have to have somewhat stricter communication with the customer. […] We noticed that quite strongly at the moment when the people, our customers, just no longer had, I say, the normal everyday communication about jobs or about social events, they have tried/many have tried to compensate for this social need […], a lot of private conversations/a lot, a lot of things, where, yes, people who don’t want to wear their masks, people who are dissatisfied with the political situation and where one then also had to separate quite rigorously between service in the store and yes, private needs. […] And that is more difficult to achieve for people with disabilities, […] it is much more difficult for someone who wants to help the customer more, where helping, the need to help, is in the foreground.*”

(Supervisor #11, male)

In other sectors such as gastronomy, daily work routines had changed and the workload had increased. More time was needed to serve customers.

“*Of course the daily work routine has changed […] now to ensure such a, such a good customer throughput, is of course also difficult, because you also need more time to serve the guests, […] and that means of course a high workload.*”

(Supervisor #5, male)

### 3.3. Personal and Work-Related Impacts of Social Firms’ Employees during the COVID-19 Pandemic

#### 3.3.1. Fears of Infection with SARS-CoV-2

Overall, supervisors reported that there were health concerns for some employees who had pre-existing conditions when being in contact with guests. In some cases, it was also expressed that hearing-impaired employees were most fearful of infection. At the beginning of the pandemic, employees also wore face masks outside with sufficient distance and changed gloves more frequently due to fears of infection.

“*In fact, so that was, the hearing-impaired staff of mine, […] they were actually the most scared with the whole Corona time.*”

(Supervisor #12, female)

“*So with people who have a pre-existing condition, and that concerns actually almost all, […] who actually also fall into this risk group, who were then already afraid at the beginning, perhaps also to infect guests or so also when the work started again.*”

(Supervisor #5, male)

Supervisors and employees also reported concerns about being infected while commuting.

“*One or the other then comes in the morning with the train and then tells, there were already some without a mask or so, yes. From this you can see that they are all concerned with it and are also a bit afraid and so.*”

(Supervisor #2, male)

Employees noticed nervousness and sensitivity in colleagues during the pandemic.

“*I feel everyone is nervous like that and then maybe you’re […] hypersensitive then to other things.*”

(Employee #6, male)

In addition, one employee expressed her uncertainty regarding the correct use of hygiene rules at the beginning of the pandemic. As she knows how to protect herself properly, she is doing better.

“*So at the beginning it was difficult for me […]. I was more afraid and so on. Because I didn’t know exactly how to do it hygienically. But now I feel better since I know how to protect myself.*”

(Employee #5, female)

#### 3.3.2. Implementation Challenges of Hygiene and Distance Regulations during the COVID-19 Pandemic

The implementation of hygiene and distance regulations was accompanied by many challenges. In general, situations were expressed as challenging to wear a mask over a long period of time or when it was warm, when sweating, when the face was touched more often with a mask, when they had to be changed regularly, when employees and supervisors had to walk a lot or when glasses were fogged.

“*Yes, actually quite well, but of course there are also situations where it is very difficult, especially with the wearing of masks, when it is so warm and when you walk around a lot.*”

(Supervisor #2, male)

“*Yes, and always wear a mask, it’s hard to breathe and it gets damp from the moisture produced by the machines. And then it’s so hard to breathe. It’s pretty bad to wear a mask all the time.*”

(Employee #8, male)

“*Working with a mask is really hard.*”

(Employee #9, female)

One employee also explained that wearing a mask at work made it more difficult to understand customers audibly and that this potentially led to mistakes.

“*We have masks and the guests also have masks […]. And that’s why at the moment, when the kitchen is loud, we always say, ‘Please be quiet, please be quiet’. Because we don’t understand a word of what the guest is saying. At the beginning we also made mistakes a few times because they/we didn’t hear properly what they were saying.*”

(Employee #14, male)

Particular challenges resulted for hearing-impaired employees when everyone had to wear a face mask because they were not able to apply speechreading.

“*That is of course also problematic for one or the other, just like my hearing-impaired colleague, which is of course difficult when he wears a mask, so when we wear masks and he doesn’t understand us, because he can read lips, that’s of course an obstacle that we have to overcome.*”

(Supervisor #12, female)

It was also reported by employees that the COVID-19 restrictions at work were in conflict with the working conditions and premises of staff. The distance between employees in premises was not always met because their work required contact between employees.

“*We can’t keep any distance in the dish room because it’s way too tight, way too narrow. […] we are actually too close to each other, but there is no other way because the machines are too/because it is too narrow.*”

(Employee #8, male)

Partly, employees got used to the pandemic-related situation, although a tiredness due to high frustration levels were described among employees. Therefore, supervisors needed to convince employees about COVID-19 related consequences and hygiene and distance regulations also when in contact with costumers.

“*We have now slowly become accustomed to this situation, although I observe that some employees are too relaxed in dealing with the crisis and need to be reminded once again to wear a mask when in contact with customers and to comply with the regulations. It can be observed that a fatigue is developing and that it is difficult to combat this, because there is now also such frustration, especially among employees, who are also saying, yes, what’s the point, everything has been shut down, there is nothing left, so it is also becoming more difficult to argue against this.*”

(Employee #8, male)

Other employees also forgot hygiene and distance regulations and had to be reminded, e.g., when employees hugged each other (which especially applied for outsourced sheltered workshop employees).

“*So they come now and then and just hug you or hug each other and […] there you already watch out that they do not do it.*”

(Supervisor #7, female)

#### 3.3.3. Sudden Loss of Work during the COVID-19 Pandemic

The pandemic-related restriction of social contacts in spring 2020 resulted in a temporary closure of the company, which was experienced by the majority of employees as very stressful.

“*Yes, the day we had to close completely. That was first shock and then sadness.*”

(Employee #12, male)

“*Yeah, that’s the thing. When you’re at home, you miss everything. The work we missed, we always called […]. The first time we started after Corona at [name of workplace], I was the first.*”

(Employee #14, male)

Some employees highlighted the boredom they experienced as a result of not being at work during this time.

“*Where/not to work, I’ve got cabin fever.*”

(Employee #12, male)

“*Without work or nothing at home that always extends the time […] is not nice.*”

(Employee #10, female)

Similarly, supervisors stayed in contact with employees during lockdown and reported that boredom was a problem during short-time work.

“*I had contact with my target group employees mainly, they just kept saying they were bored, so that was the biggest factor actually, that they were bored.*”

(Supervisor #7, female)

Some employees stated that their experience of stress could be stabilized again over time.

“*In any case, it’s going better now compared to the beginning.*”

(Employee #14, male)

“*And (…) well, and now with the work I’m a bit calmer again. The salary is back to normal and so on and so forth. But (…) that was already bad.*”

(Employee #6, male)

Other employees, in turn, were also able to name positive aspects of the lockdown, e.g., less stress at work or more free time, but these again resulted in negative impacts over time. One supervisor also noted that employees found it positive to be able to spend more time at home.

“*The first two or three days or weeks were of course relaxed. Then you thought, ‘Yeah, okay, it’s cool. You’re at home.’ but I think after a while I got annoyed. And then I also thought, ‘Come on, I want to go back to work, because you can’t really go out.’ You can’t kind of do anything outside now as well.*”

(Employee #11, male)

“*Also kind of good sometimes that only a few were there. For example, after cleaning we were sometimes allowed to go home and that was good […]. But what was bad was that there wasn’t much going on, that you didn’t see any more people, that (name of city) was dead […]. And hopefully never again this lockdown, that’s been really bad.*”

(Employee #3, female)

“*I think for me it was also unfamiliar, the situation: uncertainty, completely at home, you can’t go out, you can’t do anything with friends, you can’t go to clubs or something. And/yes you also didn’t know when it would continue. And/I think you can already feel a certain difference. […] some is just through Corona—as it was at the beginning—less stress, just a little bit. And now slowly the stress is more, that’s how I feel sometimes.*”

(Employee #7, male)

“*So on the whole, they all think it was great that they were at home.*”

(Supervisor #11, male)

#### 3.3.4. Conflicts When Working in Different Departments during the COVID-19 Pandemic

Some supervisors described that the restart in May 2020 resulted in conflicting situations, because colleagues worked at different workplaces than before:

“*So let’s say colleagues have reopened with us in the (name of the company), which we all found very strange […], suddenly completely different people have worked there, which has also led a little to bad mood in the team […]. In such cases, of course, you have to plan a little bit according to social aspects, […], it was not considered who needs the money urgently, who should be the first to start working here.*”

(Supervisor #5, male)

#### 3.3.5. Deviation from Everyday Life/Lack of Daily Routines during the COVID-19 Pandemic

Supervisors highlighted that the workplace in a social firm provided employees a routine from which it was defined as challenging if it breaks away for a longer period of time. In the case of employees with stress disorders, for example, every new process differing from everyday life has consequences for their acquired stability.

“*It’s clear that when you work with people with overload disorders, for example, that every new process that is a little different from everyday life naturally also leads to problems and naturally also leads to additional work, because they are naturally first introduced to the whole topic accordingly. People are also much more likely to have fears and irrational fears, which then have to be dealt with additionally. These are factors that naturally come into play, of course.*”

(Supervisor #11, male)

Additionally, for employees with addictive disorders it was a problem to work only every other week, because in the non-working week there was no structure and a risk of relapse. In general, supervisors felt they are able to identify demanding situations of employees, changes in their demeanor or when they had stopped taking their medication at early stages, which was not possible during the pandemic.

“*There are also people with addiction problems, for whom it is also a problem to work only every other week, because in their non-working week they then also lack the structure and the, so there is then also a risk of relapse, […] these are just such a bit additional problems that are there and there are of course also people who simply do not get along without money, relatively many who have to apply for supplementary benefits and see how they now make ends meet, yes.*”

(Supervisor #5, male)

In other social firms, some hospital stays of employees were recorded without knowing the exact motives, although an association with a missing daily structure and loneliness in the case of frequent staying alone at home was assumed.

“*I know from two that there was a hospital stay. But I only know that. I don’t know any motivations. But I assume that’s what it’s due to […] and these were also people who were not there for a longer time or had to work under unaccustomed conditions and I think there is, there is actually exactly the case. That the structure of the day has broken away and that simply has a lot to do with it, so that one is much too much alone, because one is much at home. For many here, the workplace is also where they see people and look forward to people and so. But there I would know now, so offhand I can only think of two cases. And by ‘only’ I don’t mean that in a pejorative way at all, I’m really grateful for that, because that could have been quite different.*”

(Supervisor #14, male)

One employee felt that he was better able to maintain his performance level if he had a daily routine at work. Changing his working hours through short-time work also allowed him more free time, but he also experienced himself as more inactive due to weekly changing working times.

“*But if you work for a week and take a week off, then you’re also tired at home and so on and you’re even more exhausted from this week that you’re taking a break than if you’re working all the way through it. Because somehow you have such a working level and such a routine that you/that it doesn’t matter. But if you take a break now, […] it’s nice, because you can mow the lawn at home or do something else. But yes, it’s also exhausting.*”

(Employee #8, male)

Therefore, social firms tried to re-engage employees based on individual preferences and social firms’ orders. In general, supervisors tried to keep an overview of what has been changed in the past, what will be changed in the future and in what steps it will be done.

“*Stability at work is/the stability for many/for many people in their lives. And if you shake something, if you turn some screw, then you simply have to have in mind what kind of screw you are turning in people’s lives. For one person it’s a small thing, for another it’s a big thing. You always have to look a little bit. That’s why we try to keep an overview, what have we changed in the past? What do we want to change in the future? And how do we do that in the/in the smartest steps?*”

(Supervisor #14, male)

#### 3.3.6. Additional Work during the COVID-19 Pandemic

The COVID-19 pandemic has resulted in additional work for staff (especially in the cleaning and gastronomy sector), both due to increased disinfection needs at workplaces and reduced staffing per shifts. This circumstance was experienced as stressful and health impairing.

“*Yes, cleaning is also a bit of a difficult topic, because there simply aren’t enough workers to constantly clean everywhere, of course the tables are wiped down and disinfected, but there could be a bit more going on.*”

(Supervisor #5, male)

“*The companies that was forced on them, that we had to clean up more so that everyone stays healthy. And of course we didn’t like that we had to clean so much. And of course we had health problems as a result.*”

(Employee #2, female)

“*I’m pretty overworked at the moment since Corona. Before, there were two of us working with [colleague’s name], we always had two shifts and it worked better then. We were even three, because we still had (…) another colleague […]. So it was better then. Now there are fewer of us.*”

(Employee #8, male)

Companies that experienced an economic upturn during the pandemic also reported that while employees were happy to have something to do, felt at the same time strained and stressed.

“*And some employees were also quite stressed, […] many are then also happy that they have something to do again, at the same time the stress factor increases, so, double-edged sword.*”

(Supervisor #8, male)

#### 3.3.7. Lack of Movement during the COVID-19 Pandemic

Due to short-time work and the pandemic-related restriction of social contacts, employees learned to appreciate exercise and wished for their daily work routine to return.

“*But from, from work, ‘Yes we have become so fat, we can’t move anymore, we want to get out, we want to work’, so that came from all sides.*”

(Supervisor #4, female)

“*In the past, people always thought: ‘Oh no, I don’t want to go outside. What should I do outside, I’m bored, I’d rather stay at home’. But in that time you’ve really learned that being outside, moving around freely at all, is really a lot.*”

(Employee #11, male)

#### 3.3.8. Lack of Social Contacts/Loneliness during the COVID-19 Pandemic

In social firms, the workplace represents a routine for its employees with high levels of social benefits and was therefore described as challenging when social contacts were not maintained over a long period of time. Colleagues were even compared to a family providing a good social environment and high levels of cohesion. Employees worked less for the money and rather to get out and learn new things.

“*So actually the social aspect is more important with the people, because they are here like in such a family, they are caught up, if what is so of course personally and private problems we cannot regulate, but they see each other every day and are happy and have such a, good social environment, almost friendly and that’s what they missed the most.*”

(Supervisor #4, female)

“*Yes, family is good too, but work is fun. When you’re here eight hours, we’re all more than family, work. For example, I see [name of a colleague], I see him eight hours a day. But I don’t see my kids that much. My wife, two hours. I see/am with [name of a colleague] eight hours.*”

(Employee #14, male)

“*I think it was also a bit of a loneliness issue, especially in the 100 percent short-time work, that simply the structure, that, or the, the daily structure was simply broken and many were at home and yes, simply no longer had their daily work routine, which was already very difficult for some.*”

(Supervisor #13, female)

“*That was […] Monday before the complete closure, there was again/there was such good humor and I totally miss the good mood.*”

(Employee #12, male)

One employee only realized how important social contacts are for him due to the pandemic-related contact restrictions and short-time work.

“*You weren’t allowed to meet with other friends or anything that was just so strict. You thought, ‘Ah okay, we’re missing something here.’ But what I learned from that is how much we actually need this/contact with friends. So going out, even if you really don’t do anything outside, that you just go out, meet a colleague, just talk/or maybe go out alone. Because it was really/the first months had only been at home. And that’s when you really noticed how important it is for us to go out and see what we’ve got/how/how good we actually have it here, right?*”

(Employee #11, male)

Despite the challenges of the pandemic, some employees reported that they continue meeting with colleagues on free time in summer 2020.

“*Now in Corona, we go to the restaurant, private, food. Too bad that now we cannot go to the disco. We also went a lot of dancing with colleagues. […] Yes, this Saturday again. […] We do a lot together.*”

(Employee #10, female)

#### 3.3.9. Financial Challenges during the COVID-19 Pandemic

The temporary closure of the company and the shift to short-time work was experienced by several employees as a heavy financial burden. One employee described his job as one of his basic necessities of life, which had been lost during restriction of social contacts as a result. The associated financial uncertainty as well as uncertainty regarding social firm’s future developments were described as very stressful.

“*One of my basic necessities of life is work. That’s just broken away. Then the salary just dropped off. Total uncertainty. I didn’t know how/will our store open again? How it will/So it was totally awful.*”

(Employee #6, male)

“*Without salary either. We got less. We had to make a lot of phone calls, do a lot of walking to get it all back. A little bit. It was difficult for two months and then it’s back on again.*”

(Employee #14, male)

“*My wife earns well and I have sent all the documents to the authorities and there is no/no relief. We built [a house] and so on. Of course, you have to make sure that you pay your bills on time and so on. And that hasn’t, hasn’t been fun.*”

(Employee #6, male)

“*Yes, so I’m making a loss because of Corona. I only get my 85% and what I work with/I make a loss every month. Corona is really expensive for me. Because I don’t get any support, because my wife earns so well. […] That’s what’s missing, the money.*”

(Employee #7, male)

Supervisors also reported that many employees faced financial constraints and challenges when applying for supplementary benefits, as well as existential fears and housing-related challenges. Therefore, supervisors supported them, e.g., by downloading forms or filling them out together, e.g., due to language problems.

“*In the time when we didn’t work at all, well I also kept contact with the people, I also have employees who were partly overwhelmed with applying for supplementary benefits […] where I actually also provided support, so I downloaded the forms on the Internet, sent them to them, or I filled out the forms together, so that they could apply for these benefits at all, because […] they are simply linguistically already overwhelmed with it.*”

(Supervisor #5, male)

“*Another problem was, employment office had said, we don’t want so much documents in Corona period. But after that, they wanted a lot of papers […]. For short-time work, we needed a lot of paper […] I don’t think that’s good in this period!*”

(Employee #9, female)

Employees were initially worried that they will not get enough money and thought about how to top up their benefits, e.g., at the employment agency. Supervisors were partly able to allay fears for which employees were grateful.

“*They were first afraid […], that they do not get of course, exactly too little salary and have of course thought about how to top up, whether at the employment office or something else, but after I have then taken away their fears […], they were rather positive and very grateful.*”

(Supervisor #12, female)

Additional granting of private loans for employees was outlined by supervisors.

“*I have also given private loans at the time, so as a transition to the people, so that they have come at all in the time when the money was then suddenly quite scarce, that already, so I think that there also, there were financial bottlenecks, certainly also existential fears.*”

(Supervisor #5, male)

#### 3.3.10. Fears of Job Loss during the COVID-19 Pandemic

Supervisors outlined employees’ fears of job loss. In some cases, financial security could be intercepted quickly and communicated to employees. In addition, supervisors described that employees in the catering sector reported fears when staying at home over a long period of time without any political changes and when hearing about closing companies.

“*Mainly fears that they will no longer be able to work later. That the company would just go bankrupt. That was the main fear of people.*”

(Supervisor #16, male)

One employee also reported insecurities in the team regarding their jobs, as he had experienced how positions were not being reoccupied. Thereby, he recognized the financial difficulties of the company.

“*Right now at work, I see everybody kind of struggling with their salary, like everybody’s struggling with Corona. I don’t want to leave anybody on the team or anything and the people that once did that, they’re somewhere else or they’re not on the team anymore or they’ve left and there’s nobody kind of followed up. […] Yes, above all, you have to see where you can hire in Corona/who you can hire now or so, because you don’t know where it’s going. I would not want to change places with the head of the company, because he doesn’t get his salaries for free. He has to calculate cold as ice, but for him (…)/I don’t know what to do.*”

(Employee #6, male)

On the contrary, other supervisors expressed that the pandemic was not the main topic in conversations or that no existential components were experienced because the social firm was located in a sector described as a crisis winner when taking sale numbers into account.

“*Since we don’t have these existential components, because as I said, we are just, if you like, crisis winners—no.*”

(Supervisor #11, male)

Individual supervisors described that they provided accessibility in the office via mail or messengers, but those offers were not used by employees. The reason given for this was that employees do not experience any fears.

“*Rather not, because they were sent home and then we communicated, so to speak, only by e-mail or also by telephone via WhatsApp, which was, I did make that available, that I could be reached here in the office, but that was not used.*”

(Supervisor #8, male)

### 3.4. Personal and Work-Related Impacts of Social Firms’ Supervisors during the COVID-19 Pandemic

#### 3.4.1. Fears of Infection with SARS-CoV-2

Individual supervisors highlighted that they felt uncomfortable when being in contact with customers in the salesroom, wherefore it was discussed to avoid certain work tasks such as counselling.

“*And I go very reluctantly upstairs […] my main room is the workshop and not the sales room, only in most extreme need, most extreme need, I go then just times up there. […] I would like to avoid it quite at work of course. Great contact.*”

(Supervisor #9, male)

#### 3.4.2. Dealing with Less Staffing and Interception of Work during the COVID-19 Pandemic

Challenges for supervisors were highlighted in summer 2020 when working fulltime, in two teams with less staffing and when intercepting work-related stress of employees.

“*Because we are economically a bit tightly knit due to Corona, we have just now noticed that there are actually conflicts because the kitchen staff is simply understaffed […] and simply stress arises because of that and on the other hand, that is also compensated for by more work by other people.*”

(Supervisor #13, female)

Traced back to less staffing, challenges were also reported when dealing with administrative work.

“*At the moment, it’s not possible with the small teams anyway, because there are still tasks to be done in the afternoon that the others can’t do, such as cash accounting or the like, so you’re still there until the end […]. So administration I do primarily from home, […] set are actually two hours a day or one day a week, I did that for a while, when we worked in a full team and I have my deputy, who can then take over the thing on the day, then I go out sometimes a day, now a long time no longer.*”

(Supervisor #5, male)

Likewise, individual supervisors reported that they worked alone because of low customer volumes, or that they changed workplaces because former departments were temporarily closed during the pandemic.

“*Nevertheless we are halved in terms of personnel, because we form two teams, which means that only half of the people work for a week, so that leads to a bit of stress at the moment.*”

(Supervisor #5, male)

#### 3.4.3. Economic Impacts for the Social Firm during the COVID-19 Pandemic

Furthermore, the economic situation was mentioned as challenging, as it was mentally upsetting when receiving cancellations, especially as a small company.

“*With me personally? Well, now here the economic situation perhaps, […] so it’s mentally hard when the cancellations come in and you know that thousands of euros that were planned don’t come in, so that, yes, but in the end, yes, I can live with that, even in my function.*”

(Supervisor #12, female)

#### 3.4.4. Conflicts When Working in Different Departments during the COVID-19 Pandemic

Other individual supervisors reported due to short-time work and assignments at different workplaces that they did not longer know where they belong and had to learn new work tasks and flows. Conflicting situations were stated between the supervisors in terms of hierarchy.

“*Exactly, you don’t know where you belong. Colleagues who are actually colleagues sometimes behave as if they were superior to you and, because you are in their department and yes, you always have to learn new work, learn new things again and again, because you don’t know the companies either.*”

(Supervisor #7, female)

#### 3.4.5. Additional Work during the COVID-19 Pandemic

Due to a change of personnel in the social firm, new tasks were assigned to supervisors, e.g., in administration, which was outlined as a challenge because it had to be done in addition to regular tasks. Due to the pandemic there was some time to get used to it.

“*I would say that it is challenging because [someone] has left and we have been assigned his tasks, so to speak, to us, i.e., to administration, which is a great challenge at first because you have to do everything on the side, but it is not every day […] and therefore we have a bit of time for these things, so, no, that, we now have time to get used to it*”

(Supervisor #4, female)

Overall challenges for supervisors included extra work for them because employees had to be introduced to the pandemic-related measures and they were more likely to have irrational fears that supervisors had to deal with. Supervisors in social firms were considered to play a supportive role for employees with disabilities in many ways. Thus, supervisors reported receiving many calls from employees who were unsettled.

“*Of course we also had a lot of calls from participants who were insecure and so, that all had to be intercepted.*”

(Supervisor #1, female)

#### 3.4.6. Challenges in Detaching from Work during the COVID-19 Pandemic

Difficulties in detaching from work and a lack of opportunities for stress relief, such as conversations with friends, were reported as increased stress levels for the supervisor.

“*Corona has really made my workplace much more stressful. And the compensation possibilities, so what used to help—before Corona—was to simply turn off the head, or fill it with other things. For example, talking to friends […] and those just don’t exist anymore. And I miss this, this distance from work.*”

(Supervisor #14, male)

#### 3.4.7. Fears of Job Loss during the COVID-19 Pandemic

In general, the situation was stated as a shock by supervisors, due to uncertainty, depressed moods and little compensation at the beginning of the pandemic. Other supervisors described themselves as free of fears and worries about becoming unemployed due to a good personal network.

“*So the first three weeks I was very depressed […], I would almost say, so it was no longer depressed, but it was really such a phase where I thought, ‘Oh God what’s going to happen and will the store be closed’ and, so I had strong fears of the future.*”

(Supervisor #4, female)

I’m relatively free of fears and worries when it comes to that, because I always find work.” (Supervisor #16, male)

#### 3.4.8. Positive Side-Effects of Work-Related Changes during the COVID-19 Pandemic

Positive side effects were also described by supervisors at the beginning of the pandemic, such as enjoying time with family, doing activities there was otherwise no time for while working (e.g., wallpapering, painting) or that stressful work weeks could be followed by a week of regeneration. Additionally, the pandemic also reduced private stress of supervisors, so that it was possible to detach from work.

“*For me personally, of course it was a bit of a shock at the beginning, now we’re all staying at home and what we are going to do now and how will it all develop. But I must say, for myself the Corona time where I was with my family at home was actually also very nice. No, so we had breakfast together, lunch together and, which you don’t have in normal everyday life, because one is working, maybe at school or something like that.*”

(Supervisor #2, male)

“*At the moment, of course, it’s okay, because you only work 50 percent anyway, you always have, when you have a stressful week, you always have a week where you can regenerate and shut down again.*”

(Supervisor #5, male)

To sum up, Table 3 provides an overview of work-related changes in social firms due to the COVID-19 pandemic and associated personal and work-related impacts for employees and supervisors.

## 4. Discussion

The present study was one of the first considering the setting of German social firms by using a qualitative approach to examine occupational changes as well as personal and work-related impacts for employees and supervisors during the COVID-19 pandemic. According to the proposed research questions, sector-specific economic changes, the introduction of short-time work, the implementation of hygiene and distance regulations as well as the adaption of personnel planning strategies and cooperation and distribution of work were presented as a result of the COVID-19 pandemic. Differences on personal and work-related impacts of employees and supervisors were displayed. While employees reported implementation challenges of hygiene and distance regulations, sudden loss of work, deviation from everyday life and missing daily routines, a lack of movement, social contacts and loneliness as well as financial challenges, supervisors were confronted with less staffing and interception of work, challenges in detaching from work, and a strained economic situation. Likewise, participating supervisors also underlined positive side-effects during the early stages of the pandemic. Both target groups informed about fears of infection with SARS-CoV-2, conflicts when working in different departments, additional work and fears of job loss.

### 4.1. Work-Related Changes in Social Firms during the COVID-19 Pandemic

Most of the social firms were closed completely in between March and May 2020, followed by a phase of reopening. As a result, decreased sales were observed during the year and short-time work was implemented. As described and in line with our results, sales declines mainly affected the catering and hotel sector, as well as education and entertainment. Most of the companies had to rely on short-time allowance, other subsidies and financial support services [16]. However, in the first place, social firms in many states did not have access to emergency aid for the economy and due to their non-profit legal form, these companies were not able to accumulate financial reserves. In July 2020, bridging aid for small and medium-sized companies were introduced also considering non-profit social firms. An additional EUR 100-million program was set up in early 2021 providing additional support [15].

When restarting business operations, hygiene and distance regulations were implemented, which were largely accepted among employees, although accompanied by some challenges, such as colleagues not adhering to those regulations. As depicted by several authors, people with disabilities may have difficulties understanding and complying to hygiene and distance regulations (e.g., physical distancing, handwashing or wearing a face mask), e.g., for those with mild intellectual disabilities [30,31]. Additionally, people facing visual or sensory disabilities may need to depend on more haptic impressions (such as the use of assistance or touching public spaces for navigating), which may affect infection prevention [31]. In line with our results, hearing impaired individuals were confronted with communication barriers while using face masks, which prevented lip reading [31,32]. Furthermore, a lack of information about the disease, measures for infection prevention or health services may be faced by people with sensory or intellectual impairments. People with mental health conditions may be exposed to symptom worsening and an elevated risk of severe psychiatric morbidity and those with physical impairments may experience functional deteriorations [31,33].

### 4.2. Personal and Work-Related Impacts of Social Firms’ Employees during the COVID-19 Pandemic

Within our study, several participants stated health concerns and fears of infection when being affected by pre-existing conditions, in contact with guests or during commute. Results are supported by a study conducted in the Netherlands, informing of substantial worries and anxiety of people with intellectual disabilities during the initial stage of the pandemic [34] and from a Canadian study outlining increased health concerns of employees with both physical and mental health disability compared to non-disabled participants [35]. Likewise, diverse consequences and coping behaviors of people with intellectual disabilities were stated, including emotional upset, fears and anxiety, an increase in depressive symptoms, social withdrawal, restlessness, aggressive behavior as well as uncertainties and helplessness [17,18,19]. For people with severe mental health conditions a decreased ability to deal with lockdown induced stress as well as an elevated risk of relapse was stated in the current state of research, which also supports the present results [31]. Several authors [30,31] summarized that people diagnosed with autism spectrum disorders faced emotional challenges and anxiety, e.g., during self-isolation or when practicing physical distancing due to changing habits. Furthermore, they might be too concentrated on and in turn overwhelmed by pandemic-related information on different media, its risks and measures for infection prevention, which may increase anxiety and paranoid thinking patterns. Additional disrupted behavior due to lacking routines may be observed.

The temporary closure of the social firm and sudden loss of work was experienced by the majority of employees as stressful, especially when being confronted with a lack of routine over a longer period of time without the provision of the company’s social benefits. The adaption to new processes differing from everyday life were described as having consequences for employees’ acquired stability. For instance, those with addictive disorders faced a higher risk of relapse in non-working weeks and hospital stays were assumed to be linked to missing daily structures and loneliness. In line with our results, Habermann-Horstmeier (2020) also reported about lacking routines for people with disabilities, adapted working conditions, reduced social contacts, a loss of appointments, leisure activities and fewer contacts with professionals in several fields, like education or psychology [17]. In general, employees with a physical and mental disability claimed significantly less organizational support than those having no disability. Employment conditions were summarized as relevant influencing factors of COVID-19 perceptions of disabled employees [35].

When working in social firms providing basic routines and fitting work accommodations, several studies indicated positive health-related outcomes on multiple levels for employees such as well-being or the promotion of recovery from mental health conditions [7,8,9,10,11,12,13,14,36,37]. More detailed, results from the UK highlighted a reduction in hospital visits, self-harm, less contact to counsellors or psychotherapists, psychiatrists, or crisis teams and less medication intake [9]. Accordingly, the absence of supportive working conditions in this particular setting could be assumed to have an impact on employees’ health. In fact, the situation of people who already suffer from depression has increasingly deteriorated as a result of measures for infection prevention [38,39], (e.g., 44% of people diagnosed with depression reported a worsening of their illness in the last 6 months, up to suicide attempts [39]). Likewise, data of a meta-analysis indicated an increase of symptoms of anxiety and depression in the general population during early stages of the pandemic [40] and elevated levels of loneliness and social isolation [41]. Referring to the latter, people with a disability were exposed to an elevated risk for social isolation and loneliness even before the COVID-19 pandemic, resulting in a vulnerable pre-pandemic situation [4]. As a consequence of social isolation and loneliness, research indicated an impact on different physical and mental health dimensions including, e.g., elevated systolic blood pressure and an increased risk for heart diseases, dementia, a higher all-cause mortality, sleep efficiency, depressive symptoms, poorer self-rated health and associations with suicide (attempts). Moreover, behavioral changes were reported comprising lifestyle-related aspects such as poorer dietary behavior, smoking, alcohol consumption, physical activity or adherence to medication intake (e.g., [42]). Recognizing the links between work and its relevance for the target group and health-related outcomes can help to develop effective interventions for future pandemics or when facing higher incidences, e.g., due to new virus variants.

Current results included not only a lack of routines and social contacts for employees in social firms, but also affected their physical activity, which is why exercise and daily work routines were appreciated. Additional recent analysis in two reviews identified decreased physical activity also as one of the major topics affecting people with disabilities during the pandemic [31,43]. In fact, being less physically active during lockdown was associated with having a disability, wherefore it was suggested to consider policies on improving physical activity during lockdowns for vulnerable groups [44]. Consequences on being less active during lockdown were further analyzed in a study from Germany, according to which 40% of the 1001 respondents (regardless of the disability status) have gained weight since the start of the pandemic and were also less active than beforehand (the higher the respondents’ Body-Mass-Index (BMI), the more often they have gained weight [45]).

As an additional component during the COVID-19 pandemic, financial challenges of employees in combination with uncertainty regarding social firm’s further development were exemplified. Likewise, employees felt overwhelmed when applying for additional benefits, wherefore support was provided by supervisors. In general, many people with disabilities were described as in financially precarious situations before the onset of the pandemic, resulting in a deterioration of economic hardship due to COVID-19 [4]. Results from the UK support the depicted tendencies: Disabled participants were more likely to work reduced working hours and to report about financial challenges during the first three months of the COVID-19 lockdown compared to non-disabled peers [46]. Furthermore, many people with disabilities faced job losses during the pandemic, others were furloughed for an indefinite period of time and they were not offered to work remote, while small businesses and non-profit organizations were frequently closed [31,47]. Also people with cognitive impairments were likely to express worries, e.g., due to finances, safety or future plans [3] and employees with both a physical and mental health disability faced significantly greater financial concerns in comparison to non-disabled participants [35]. Experiences from before the pandemic already showed high levels of support from supervisors, e.g., regarding daily life challenges such as assistance with housing, transportation, or welfare benefits, which is why a more permanent workforce was described [36,48,49]. Illustrating the importance of this type of employment, employees of social firms were able to gain financial independence with regular wages (complementing disability benefits), which promoted in turn social participation [7,11,12,14,49]).

### 4.3. Personal and Work-Related Impacts for Social Firms’ Supervisors during the COVID-19 Pandemic

Overall, the results also illustrate that supervisors faced specific challenges and job demands during the COVID-19 pandemic. Regarding their work-related impacts, challenges were explained with the overall economic situation in the social firm due to cancellations, less staffing, interception of work and conflicts when working in different departments. Additionally, exchange of information during the pandemic did not work for all those affected, as employees never met completely and elaborated workflows were suspended. In this context, supervisors had to learn and implement new work tasks and processes, take on additional tasks, requiring supervisors to communicate information to employee group several times, and also provide a support function for employees. In terms of their personal-related impacts, it was found that the pandemic was predominantly experienced as a shock. Uncertainty, depressed moods and increased stress levels were associated with fears of infection, fears of job loss or challenges in detaching from work or finding a balance to work. However, individual supervisors were unable to report any significant worries or fears. The positive aspects of the pandemic were presented, such as more time with the family or an improved work-life balance.

Preliminary research on the stress situation of supervisors or direct supporting staff working with employees with disabilities during the COVID-19 pandemic are partially consistent with present study findings. A qualitative study on the experiences and needs of direct support staff working in intellectual disability services during the initial phase of the COVID-19 lockdown in the Netherlands found, among other things, that this staff similarly had to take on new tasks and processes, experienced time pressures, and performed additional shifts, or worked longer hours due to staff shortages [50]. In addition, the emotional and cognitive impact of the pandemic was described: staff likewise reported fears of infection due to insufficient protective equipment. In general, an overwhelming amount of emotions were reported: Frustration and disappointment due to the lack of visibility in the media and by the government, a strong sense of responsibility towards people with disabilities as well as diverse emotions by working with them, such as enthusiasm, sorrow or suffering [50]. Another qualitative study reported on the situation of people with intellectual disabilities in residential facilities or workshops in times of the COVID-19 pandemic, among others, from the perspective of supervisors [17]. The study confirmed higher workload and feelings of stress for supervisors due to new challenges at work, such as the introduction of new rules, measures, staff planning, information overload and longer working hours, as well as due to the family situation regarding childcare. The first weeks after the closure of the workshops were experienced as very stressful and uncertain, as was the fear about cases of infection or consequences if the virus spread in the facilities. In this study, supervisors were able to report also positive aspects in the course of the pandemic, such as a more relaxed schedule due to the elimination of outside appointments or conferences, as well as the possibility of integrating more pedagogy into their daily work routine [17]. However, both qualitative studies are of limited comparability to the present results, as these were conducted in institutions or workshops for people with intellectual or mental disabilities and not social firms that provide employment for people with diverse disabilities on the general labor market.

Prior to the pandemic conditions, the current state of research also outlines specific job demands and resources of supervisors in social firms. A qualitative interview study on the working conditions of supervisors in German social firms revealed that balancing social and economic goals, a high work intensity, unpredictability or overtime are among these specific job demands [51]. Perceptions of employee absenteeism and staff scheduling (taking into account absences or emotional crises), conflicts caused by lower employee performance combined with high customer expectations, and socio-pedagogical activities (e.g., employees’ acute need for support during high workloads) describe the specific challenges in balancing economic and social goals [51]. Furthermore, a quantitative survey of supervisors and professionals in workshops for people with disabilities revealed that the majority had an increased risk of burnout due to job stresses such as low predictability, a low sense of community, high physical demands, and a low level of appreciation [52].

It becomes evident that leaders in social firms are exposed to specific work stresses, which have been aggravated by the current pandemic situation. Given that supervisors in general have an important role model function in occupational health promotion and have proven influence on employee health, the health of supervisors should also be systematically promoted in addition to the employees’ health [53,54,55,56]. A recent meta-analysis illustrates that supervisors’ well-being influenced leadership behavior and, consequently, employee well-being and performance [57]. Therefore, supervisors themselves need to be in an optimal stress-strain situation, understand occupational psychological relationships between job characteristics and mental health, and need to create possibilities for a positive health culture at the workplace [58,59].

### 4.4. Strengths and Limitations

Our study provides several strengths. First of all, the qualitative research approach was considered to be the most appropriate to obtain initial findings in a new field of research and to address the research objective [24]. Overall, triangulated methods (focus groups and individual interviews) were selected in a manner that was consistent and appropriate to the subject matter, as well as to the needs and accessibility of both sample groups (employees with disabilities and supervisors). Conducting focus groups with employees with disabilities proved to be particularly suitable for facilitating group interactions on team-related topics in a moderated setting. By using this method, employees, e.g., with mental diseases, language barriers or hearing impairments, were able to express themselves more comfortably in their familiar social environment and to participate in the discussions [23]. The research team was prepared for the use of sign language interpreters. However, they were not needed as hearing impaired employees in the sample were able to speech read and did not require sign language. For conducting interviews with supervisors, the PCI technique was particularly suitable for giving participants sufficient scope for their experiences, perceptions and reflections [26]. It was found that the anonymity created by conducting telephone interviews was helpful in establishing trust and confidence towards the interviewer. Furthermore, in relation to data analysis, all results were presented and discussed among all authors to provide transparency, accuracy and validity of the results. Direct quotes of participants were cited in the presentation of results to increase trustworthiness of qualitative results. Another strength lies in the sample size and composition, considering the exploratory nature of our study. The 16 interviews conducted were shown in past research to assume theoretical saturation [60]. Likewise, three focus groups, each with 3–6 employees, were conducted in accordance with the recommended group size and number of groups [25,61]. In addition, consideration was given to a heterogeneous sample composition in terms of age, gender, sector affiliation (typical for social firms), type of disability, and number of subordinate employees in order to reflect different experiences and nuances [23].

Yet this study has some limitations. First, given the qualitative study design and the aim to obtain exploratory findings, there is no claim for generalizability of present study results. Likewise, transferability is also restricted for target groups in different countries due to differences in pandemic-related consequences or legal frameworks. Second, the current COVID-19 pandemic had an impact on the study design. In order to protect the health and safety of all participants, a hygiene concept with very high standards and precautions was adopted for the implementation of each focus group. For this purpose, among other things, the maximum number of six participants per group was strictly followed. In parallel, two planned focus groups could not be conducted due to the pandemic-related lockdown in November and December 2020 in Germany. Future research should therefore examine pandemic-related experiences by employees from additional sectors. Lastly, it should be noted that additional potential interviewer bias on respondents’ answers and tendencies according to social desirability must be taken into account, as well as selection bias due to the sampling approach.

### 4.5. Implications for Policy and Practice

The COVID-19 pandemic impacted employees and supervisors in social firms on different levels. It was highlighted that most of the social firms were closed during the onset of the pandemic and short-time work was applied. Overall, eight recommendations could be derived based on the study results. (1) Traced back to the discussed disparities, it is recommended to consider the individual social and economic situation of employees, who are most in need of returning to work. After reopening, hygiene and distance regulations, adapted personal planning and work organization processes were introduced. Partly, fears of infection were reported among employees and supervisors. (2) Therefore we recommend to provide accessible and reliable information on the virus in different ways (e.g., braille, plain language, sign language) and on different media especially on prevention strategies [62]. Suggestions here include informative, accessible websites in plain language (e.g., [63]). The compliance with hygiene and distance regulations should be encouraged in the same vein.

Furthermore, the closure of the social firms resulted in lacking familiar everyday structures and routines as well as social isolation as described above. (3) Therefore sufficient resources have to be made available so that companies are able to support its employees, e.g., by means of (external) psychosocial counselling, ongoing social support and regular contact during the pandemic. (4) Additional attention should be paid to the situation of supervisors, who were confronted with various challenges during the pandemic, which were summarized as a double burden of economic and social demands. Particularly in view of the fact that supervisors take on a role model function for employees and that job demands of supervisors also have an impact on the health of employees [64], supervisors should also be provided with sufficient resources by ensuring enough scope for action, administrative support or a reduction of time pressure. (5) Furthermore, in order to foster employees’ health at the workplace, health-promoting structures should also be maintained and further developed during the pandemic, which should be adapted to the needs of employees. Individual behavioral-related as well as structural-related prevention strategies should be further strengthened and expanded (e.g., again by means of websites in plain language on promoting exercise, e.g., [63]). (6) As a supplement, digital (communication) offers can also be used to provide support and to help counteract loneliness and social isolation among people with disabilities. However, people with disabilities often face disparities due to lacking adaptations in digital solutions [31], which is why there is a need for further action in this area.

(7) Regarding future regulations, people with disabilities should be consulted not only during the planning and surveillance process, but also during education and evaluation of public health and policy interventions, especially in view of the given vulnerability on multiple levels whether, e.g., due to topics such as employment or social participation. When preparing for upcoming comparable crises, public health authorities should encourage the development of disability-inclusive interventions as well as economic support for employment programs for people with disabilities to adapt responses suitably [3,33,46,47]. In the past, needs for support of people with disabilities were associated with dependency followed by paternalistic approaches of politicians and providers [3].

(8) Overall, awareness should be raised for policymakers and public health authorities for the significance of work opportunities for people with disabilities on the general labor market and its influences on health-related outcomes when providing flexibility in terms of working hours and amount of work, a regular structure and high levels of social support [6].

### 4.6. Implications for Future Research

Conducting further research is crucial for improving understanding of health disparities related to populations with disabilities [65], especially as the target group is significantly underrepresented in health-related research [66]. Banas et al. [66] highlighted several challenges due to recruitment and retention, which results in lacking representation, and increasing disparities. Therefore, inclusive research strategies are needed, in which people with disabilities are engaged not only as a target group but also during the whole research process, from initial ideas and conception through to the reporting and dissemination phase [67]. Appropriate monitoring and transparent reporting of COVID-19 trends in people with disabilities is also recommended in light of current pandemic response efforts, as well as in future preparation for additional waves of infection [62]. Therefore, people with disabilities should continue to be interviewed about their experiences and needs, their suggested solutions in large scale qualitative studies [33]. Such qualitative but also quantitative studies could also assess the specific challenges and experiences depending on the type of disability. Future studies should be composed of heterogeneous samples (including people with different types of disabilities, age and gender) conducted in different countries and sectors. Accordingly, future research findings should be more fully integrated into adjustments to COVID-19 responses, as well as improving policy and practice. A review of COVID-19 responses and actions should be conducted in partnership with people with disabilities to examine whether decisions are appropriate for vulnerable populations [33]. Broad quantitative studies might also be useful in deriving generalizable research data. Yet, promoting the inclusion of standardized disability identifiers in data collection instruments is a prerequisite for such study designs [65]. Developments of evidence-based interventions for people with disabilities during pandemics should also be scientifically monitored and evaluated through participatory research actions.

Furthermore, more research is needed on the specific stress situation of direct supervisors and professionals working with people with disabilities in the workplace. Thus, future research results may improve the health situation of the affected persons themselves. Considering supervisors’ role model function and their significant influence on employees’ health [53,54,55,56], these findings may also have a positive impact on the health and work situation of employees with disabilities.

## 5. Conclusions

The presented study results revealed the personal and work-related impact of the current COVID-19 pandemic on social firms employing a high proportion of people with disabilities. Most of the companies were closed at the beginning of the pandemic followed by a phase of reopening, the implementation of hygiene and distance regulations and the adaption of personnel planning and distribution of work. Employees and supervisors were affected in different ways. Both participant groups faced fears of infection, conflicts when working in different departments, additional work and fears of job loss. Differences were identified when reporting challenges during the implementation of hygiene and distance regulation, a sudden loss of work, lacking routines, movement and social contacts as well as financial challenges for employees. Less staffing, challenges in detaching from work, or economic impacts for the social firm were exemplified by supervisors. Based on our findings, implications for policy and practice are presented. There is a need for further research on COVID-19-related consequences for employees with disabilities working in different employment opportunities and the provision of health-promoting structures.

## Figures and Tables

**Table 1:** Topics of the interview guideline for employees and supervisors of social firms.

Topics of the Interview Guideline for Employees	Topics of the Interview Guideline for Supervisors
Situation at the beginning of the COVID-19 pandemic Well-being of employees at work/short-time work Fears and worries of employees	Situation at the beginning of the COVID-19 pandemic Implementation of hygiene and distance regulations Adaptation of working conditions during the COVID-19 pandemic

**Table 2:** Participant characteristics of social firms’ employees and supervisors in percent.

Variable	Employees	Supervisors
*Gender*		
Male	7 (50.00%)	11 (68.75%)
Female	7 (50.00%)	5 (31.25%)
*Age*		
18–30	5 (35.14%)	0 (0.00%)
31–40	1 (7.14%)	7 (43.75%)
41–50	6 (42.86%)	3 (18.75%)
<51	2 (14.29%)	6 (37.50%)
*Working experience*		
Less than a year	2 (14.29%)	1 (6.25%)
1–3 years	1 (7.14%)	9 (56.25%)
More than 3 years	11 (78.57%)	6 (37.50%)
*Sector*		
Food and beverage service activities	11 (78.57%)	8 (50.00%)
Services to buildings and landscape activities	3 (21.43%)	2 (12.50%)
Wholesale of bicycles and their parts and supplies	-	3 (18.75%)
Printing and reproduction of recorded media	-	2 (12.50%)
Office administrative, office support and other business support activities	-	1 (6.25%)
*Subordinate employees*		
1–5	-	2 (12.50%)
6–10	-	7 (43.75%)
11–15	-	4 (25.00%)
15–20	-	2 (12.50%)
More than 20	-	1 (6.25%)

**Table 3:** Overview of the work-related changes and impacts of the COVID-19 pandemic in social firms.

Work-Related Changes in Social Firms during the COVID-19 Pandemic	Personal and Work-Related Impacts of Social Firms’ Employees and Supervisors during the COVID-19 Pandemic
Sector-specific economic changes Short-time work Implementation of hygiene and distance regulations Adaptions of personnel planning Cooperation and distribution of work	*Personal and work-related impacts of employees:* Fears of infection with SARS-CoV-2 Implementation challenges of hygiene and distance regulations Sudden loss of work Conflicts when working in different departments Deviation from everyday life/lack of daily routines Additional work Lack of movement Lack of social contacts/loneliness Financial challenges Fears of job loss
	*Personal and work-related impacts of supervisors:* Fears of infection with SARS-CoV-2 Dealing with less staffing and interception of work Economic impacts for the social firm Conflicts when working in different departments Additional work Challenges in detaching from work Fears of job joss Positive side-effects of work-related changes

## Data Availability

The datasets analyzed during the current study are not publicly available due to German national data protection regulations but are available from the corresponding author on reasonable request.

## Supplementary Materials

The following are available online at https://www.mdpi.com/article/10.3390/ijerph18178979/s1, COREQ Checklist.
